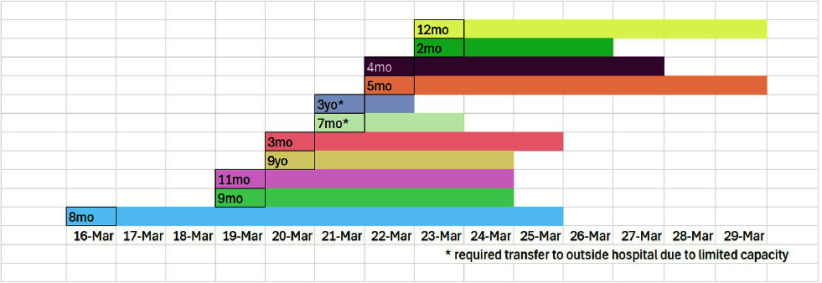# All Hands on Deck - Lessons Learned from a Single Center’s Response to a Measles Outbreak

**DOI:** 10.1017/ash.2025.375

**Published:** 2025-09-24

**Authors:** David Zhang, Rachel Marrs, Vera Chu, Camille Graham, Molly Steele, Nick Ludwig, Emily Landon, Allison Bartlett

**Affiliations:** 1 University of Chicago Medical Center; 2 University of Chicago Medicine; 3 UChicago Medicine; 4 University of Chicago; 5 University of Chicago Comer Childrens Hospital

## Abstract

**Background:** A measles outbreak associated with a migrant shelter occurred in Chicago in early 2024. Given the high transmissibility of measles in combination with the congregate nature of the shelter, health care facilities were tasked with hospitalizing patients with confirmed measles throughout the duration of their contagious period. Comer Children’s Hospital at the University of Chicago was able to hospitalize many of these patients, but numerous challenges were encountered in the initial response. **Method:** Communications were sent out to all providers to educate and increase awareness of measles presentations. Our infection prevention team helped coordinate timely collection of appropriate measles testing with the clinical team and helped facilitate timely processing with our microbiology lab for the test to be run by our state reference lab. Constant communication between the area hospitals and the city were instrumental in weathering the challenges our center faced in responding to the local outbreak. **Result:** Collaboration with the public health department allowed for optimizing turnaround times for diagnostic results, forecasting future patient volume, increasing advanced notice for patient arrival to the ED from the local shelter. Our hospital was faced with an inability to safely accommodate the influx of patients with airborne infection isolation rooms (AIIRs), and discussions with our facilities group led to the construction of multiple makeshift anterooms both in the ED and on the pediatric floors with the necessary amount of air exchanges to safely isolate these patients. A total of 18 patients were tested for measles at Comer Children’s Hospital in March 2024, including those from the community who did not reside in a shelter. Ten patients tested positive for measles, all of whom lived in the nearby shelter. Ages ranged from 2 months old to 9 years old. Patients returned back to the shelter after their infectious window was over. One patient suffered a complication of bacterial empyema requiring readmission. No exposures to patients or staff members occurred. **Conclusion:** Strong, efficient communication amongst hospital leaders allowed us to safely accommodate all the patients who presented with suspicion of measles. Working closely with the local public health department ensured optimal turnaround times of diagnostic results and increased our hospital’s level of preparedness.

**Figure:**
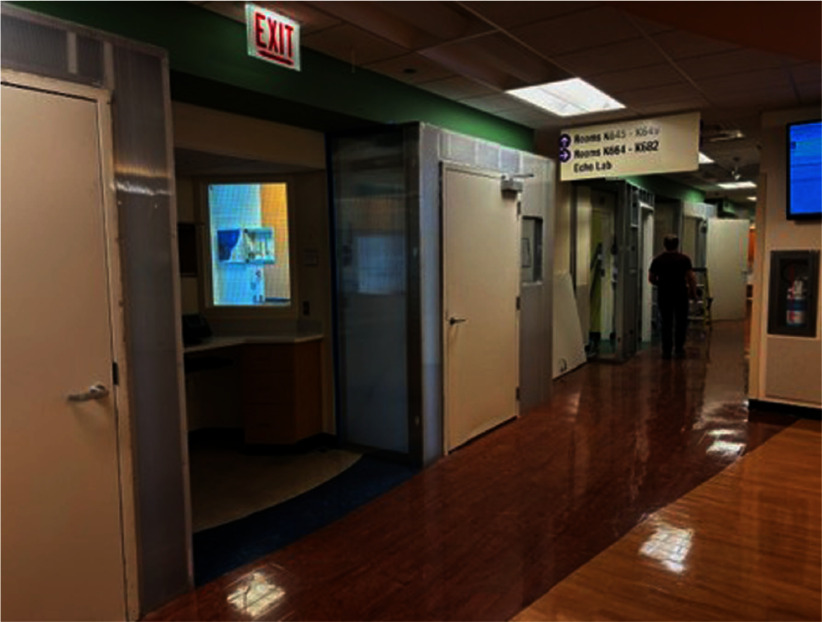

**Figure:**